# Tick-borne microsporidiosis: ticks as a neglected source of human microsporidian infections?

**DOI:** 10.1080/22221751.2024.2384472

**Published:** 2024-07-23

**Authors:** Bohumil Sak, Michaela Fibigerová, Kristína Mravcová, Nikola Holubová, Silvie Šikutová, Jana Fenclová, Martin Kváč, Ivo Rudolf

**Affiliations:** a Institute of Parasitology, Biology Centre of the Czech Academy of Sciences České Budějovice, Czech Republic; b University of South Bohemia, České Budějovice, Czech Republic; c Institute of Vertebrate Biology of the Czech Academy of Sciences, Brno, Czech Republic

**Keywords:** *Encephalitozoon cuniculi*, *Ixodes ricinus*, PCR, qPCR, vector

## Abstract

We detected 24 *Encephalitozoon cuniculi* positive *Ixodes ricinus* ticks of 284 collected in the Czech Republic. Since the route of transmission of microsporidia is not fully understood, the presence of microsporidia in ticks raises the question of whether they may be involved in the transmission of these pathogens.

Of the more than 900 species of ticks, about 10% are of medical importance to humans [1]. The life history of hard ticks consists of three stages in which they parasitize on different hosts of the same or different species. Out of approximately 70 tick species established in Europe [1], *Ixodes ricinus* is of greatest epidemiological importance in the Czech Republic. It is involved in the transmission of various tick-borne diseases, mainly Lyme disease and Tick-borne encephalitis [2]. Microsporidia are a group of unique, eukaryotic, obligate, intracellular parasitic fungi that infect insects and vertebrate hosts including humans. Microsporidia have evolved in unique and sophisticated ways that allowed them not only to survive in the environment but also to live inside other cells [3]. In humans, microsporidia were recognized worldwide as opportunistic infectious agents, mainly associated with the HIV/AIDS pandemic. However, it has been shown that microsporidiosis is not limited to immunocompromised states. Of the 17 species that are pathogenic to humans, species and genotypes of *Encephalitozoon* with broad hosts specificity have been frequently found in infected humans [3]. More recently, microsporidia have been implicated a neglected aetiological agent for more common, sometimes life-threatening diseases, such as encephalitis and meningitis in otherwise healthy individuals [4] or may contribute to aseptic periprosthetic osteolysis leading to implant loosening [5,6]. Due to the insufficient knowledge of the mechanism of microsporidian transmission, we proposed a prevalence study targeting the vector-mediated transmission of microsporidia and to evaluate the role of ticks as possible vectors of microsporidian species causing human disease.

The unfed *Ixodes* ticks were trapped by flagging low vegetation at various sites in the Czech Republic or fed ticks were collected from humans and free-living and domestic animals (Table 1). Total DNA from liquid nitrogen frozen and crushed whole ticks together with extraction negative control in each series was extracted as mentioned elsewhere [6]. Control DNA was isolated in the same way from purified *E. intestinalis* spores. *Encephalitozoon* spp.*-*specific nested PCR amplifying internal transcribed spacer [7] was used including DNA of *E. intestinalis* (PCR positive control) and ultrapure water (control without template). PCR products were evaluated by gel electrophoresis. DNA from PCR positive samples were quantified using RT–PCR amplifying a 268-bp region of the 16S rRNA gene of *Encephalitozoon* spp. [8]. Each run included unspiked specimens and diluent blanks. Results were determined to be positive when the fluorescence signal crossed the baseline at ≤43 cycles. The total amount of spores in 1 mL of individual samples was calculated based on the standard curve. PCR amplicons of the ITS region were sequenced in both directions (SeqMe, Dobříš, Czech Republic), nucleotide sequences were manually edited using the program ChromasPro 2.1.4 (Technelysium, Pty, Ltd., South Brisbane, Australia) and aligned with references GenBank submissions using MAFFT version 7 (http://mafft.cbrc.jp/alignment/software/). New batch of ticks from PCR positive locality was trapped by flagging and examined microscopically. A drop from liquid nitrogen frozen and crushed whole ticks resuspended in water was air dried, fixed with methanol and stained with standard Calcofluor M2R and examined using fluorescence microscopy [6].

**Table 1.** Structure of screened *Ixodes ricinus* tick groups, and characteristics of *Encephalitozoon cuniculi* positive samples, collected between June 2022 and April 2024 in the Czech Republic.

*Ixodes ricinus* ticks			Encephalitozoon cuniculi positive samples	
Stage	Host	Screened/positive	PCR genotypization	RT-PCR quantification
Nymph	Human	3/0	–	–
	Cat	1/0	–	–
	Flagging	68/4	1× genotype I 3× genotype II	1.1 × 10^2^ 4.0 × 10^0^; 5.4 × 10^0^; 1.8 × 10^1^
Subtotal		72/4	4	
Adult ♀	Human	24/1	1× genotype I	2.5 × 10^0^
	Cat	25/3	3× genotype II	7.1 × 10^0^; 1.4 × 10^1^; 8.4 × 10^1^
	Dog	65/6	3× genotype I 3× genotype II	1.0 × 10^0^; 9.0 × 10^0^; 1.5 × 10^1^ 2.0 × 10^0^; 1.2 × 10^1^; 1.9 × 10^1^
	Roe deer	10/1	1× genotype II	2.1 × 10^1^
	Deer	3/1	1× genotype II	3.4 × 10^1^
	Lizard	1/0	-	-
	Flagging	40/3	3× genotype I	1.1 × 10^0^; 1.7 × 10^0^; 4.7 × 10^0^
Subtotal		168/15	15	
Adult ♂	Human	1/0	-	-
	Dog	1/1	1× genotype II	3.3 × 10^0^
	Flagging	42/4	2× genotype I 2× genotype II	5.3 × 10^1^; 1.2 × 10^2^ 9.2 × 10^0^; 1.5 × 10^1^
Subtotal		44/5	5	
Total		284/24	24	

Out of 284 *Ixodes ricinus* ticks in different stages, collected by flagging (150 ticks), or from humans (28 ticks) and animals (106 ticks), respectively, we proved the presence of *E. cuniculi* in nymphs and adults of both sexes. Most positive ticks were obtained from dogs (7 samples) and cats (3 samples). A single positive tick originated from human, roe deer and deer, respectively. The most positive ticks were obtained by flagging (11 ticks). The overall prevalence among the ticks was 8.5% (Table 1). Both the negative extraction controls and the negative PCR controls were negative. We identified *E. cuniculi* genotype I in seven *I. ricinus* females, two males and one nymph and *E. cuniculi* genotype II in eight females, three males and three nymphs (Table 1, see Supplementary Figure 1). The 24 sequences obtained in this study were 100% identical to GenBank sequences of *E. cuniculi* genotype I (accession no. KJ941140) and II (accession no. MF062430) (see Supplementary Figure 1). *E. cuniculi* genotype I was most frequently detected in ticks collected by flagging and ticks collected from dogs; the number of spores ranged from 1 to 120 per tick. *E. cuniculi* genotype II was most often detected in ticks collected by flagging and ticks collected from dogs; the number of spores ranged from 2 to 34 per tick (Table 1). Microscopic analysis of Calcofluor M2R-stained smears confirmed the presence of spores (1–2 spores per slide) in two repeated samples obtained from PCR positive locality (see Supplementary Figure 2).

Microsporidiosis has been recognized as one of the most common and significant causes of chronic diarrhoea, anorexia and weight loss in severely immunocompromised patients with AIDS. Microsporidia are listed as category B biodefense agents by the National Institutes of Health (NIH) and their spores are in the Contaminant Candidate List of the U.S. Environmental Protection Agency (EPA). Recently, microsporidia have been increasingly reported to cause asymptomatic infections even from immunocompetent individuals [3] resulting in more than 4300 peer-reviewed reports, mostly represented by prevalence surveys, that focused on the detection and determination of microsporidia in various host-derived samples, primarily faeces/stool, body fluids and biopsy tissues. However, only three studies worldwide have focused on the presence of potentially human-pathogenic microsporidia in various ticks, some of which use a currently inadequate microscopic approach and describe *Encephalitozoon*-like microsporidia in *Anocentor nitens*, *Amblyomma cajennense*, and *E. intestinalis* in the *I. ricinus* tick [9–11]. In the present study, we have reported the occurrence of *E. cuniculi* in the *I. ricinus* tick for the first time. Although the PCR results could not be confirmed by microscopy due to the isolation of total tick DNA, we obtained consistent results from the corresponding tick based on both PCR and qPCR, making contamination unlikely. In addition, all negative controls used in sample processing were negative. *Encephalitozoon cuniculi* has a broad host range [12], including birds, rodents, lagomorphs, carnivores, livestock, and human and non-human primates, with the prevalence range among these hosts between 17–36% [3], and thus serving as potential source of infection for ticks This microsporidian species lacks the organ/cell specificity and infects a wide range of host cells, including epithelial cells, macrophages, kidney tubule cells, and vascular endothelial cells [13]. This versatility makes *E. cuniculi* an ideal microsporidian candidate for vector-borne infections. Moreover, closely related *E. romaleae* was detected in grasshopper, *Romalea microptera* (Beauvois) (Orthoptera: Romaleidae), suggesting that Encephalitozoonidae can multiply also in insects [14]. Rare studies addressed transovarial or transstadial transmission of microsporidia in ticks, but the assessment methodology used was appropriate for the time and therefore less sensitive, leading to negative results [15]. However, in our study, five adult male *I. ricinus* ticks (11.3%) were tested positive for the presence of microsporidian DNA, suggesting transstadial transmission, since adult males do not ingest blood meals.

Based on our results, we can conclude that the spectrum of transmission routes of microsporidia could be potentially expanded by vector-mediated transmission, providing new insights into the spread of microsporidia in animal and human populations. However, this hypothesis needs to be verified using experimental transmission studies in a future.

## Supplementary Material

Supplementary Figure 1.tif

Supplementary Figure 2.tif
