# Supplementary figures and images for: Tick-borne microsporidiosis: ticks as a neglected source of human microsporidian infections?
Source: Emerg Microbes Infect. 2024 Jul 23;13(1):2384472. doi: 10.1080/22221751.2024.2384472 (PMC11305020; doi:10.1080/22221751.2024.2384472)

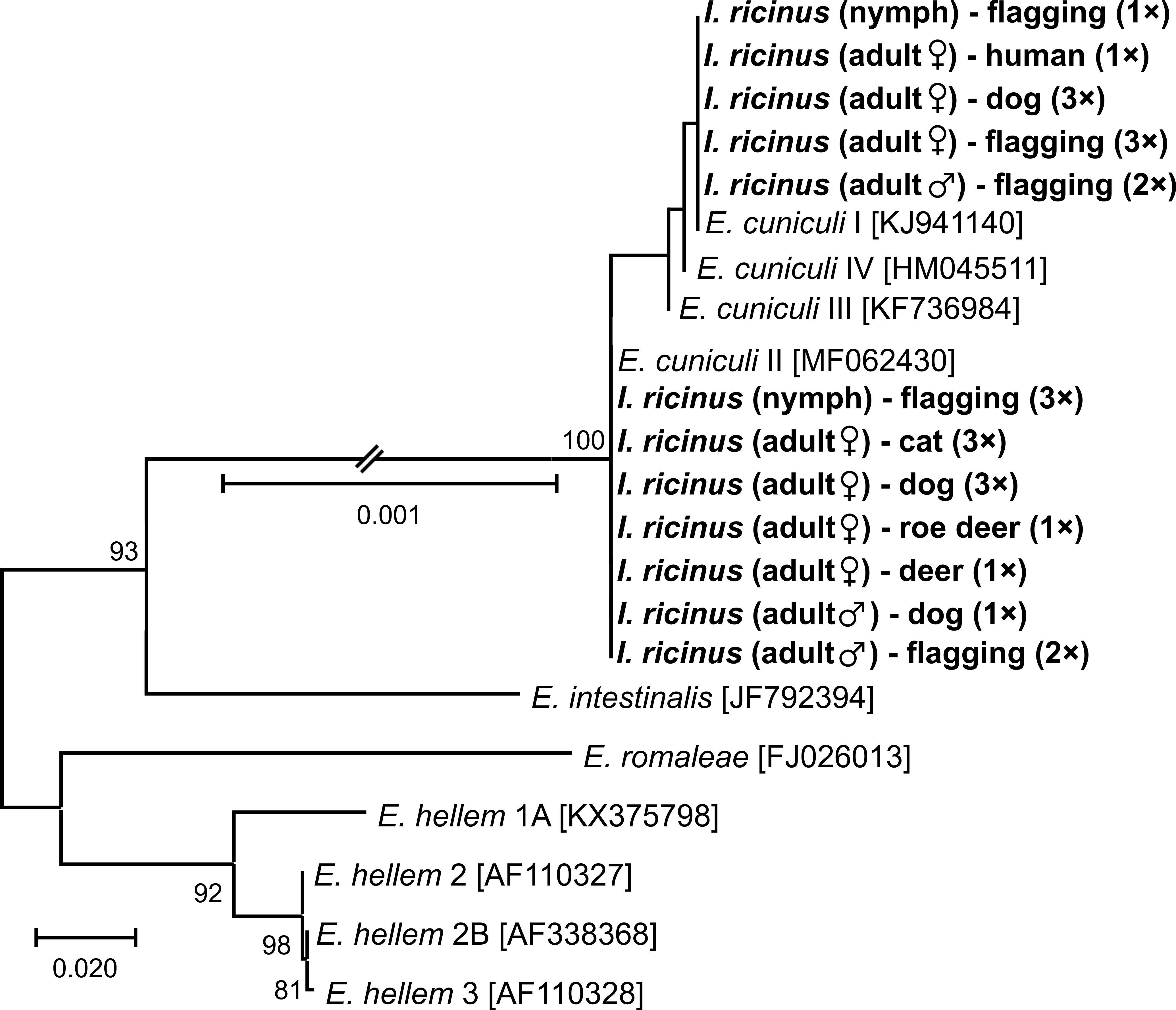

Supplement: Supplementary Figure 1.tif [file TEMI_A_2384472_SM5771.tif]
